# Recombination Accelerates Adaptation on a Large-Scale Empirical Fitness Landscape in HIV-1

**DOI:** 10.1371/journal.pgen.1004439

**Published:** 2014-06-26

**Authors:** Danesh Moradigaravand, Roger Kouyos, Trevor Hinkley, Mojgan Haddad, Christos J. Petropoulos, Jan Engelstädter, Sebastian Bonhoeffer

**Affiliations:** 1 Institute of Biogeochemistry and Pollutant Dynamics, ETH Zürich, Zürich, Switzerland; 2 Division of Infectious Diseases and Hospital Epidemiology, University Hospital Zürich, University of Zürich, Zürich, Switzerland; 3 WestCHEM, School of Chemistry, The University of Glasgow, Glasgow, Scotland, United Kingdom; 4 Monogram Biosciences, South San Francisco, California, United States of America; 5 School of Biological Sciences, The University of Queensland, Brisbane, Queensland, Australia; 6 Institute of Integrative Biology, ETH Zürich, Zürich, Switzerland; University of Arizona, United States of America

## Abstract

Recombination has the potential to facilitate adaptation. In spite of the substantial body of theory on the impact of recombination on the evolutionary dynamics of adapting populations, empirical evidence to test these theories is still scarce. We examined the effect of recombination on adaptation on a large-scale empirical fitness landscape in HIV-1 based on *in vitro* fitness measurements. Our results indicate that recombination substantially increases the rate of adaptation under a wide range of parameter values for population size, mutation rate and recombination rate. The accelerating effect of recombination is stronger for intermediate mutation rates but increases in a monotonic way with the recombination rates and population sizes that we examined. We also found that both fitness effects of individual mutations and epistatic fitness interactions cause recombination to accelerate adaptation. The estimated epistasis in the adapting populations is significantly negative. Our results highlight the importance of recombination in the evolution of HIV-I.

## Author Summary

One of the most challenging issues in evolutionary biology concerns the question of why most organisms exchange genetic material with each other, e.g. during sexual reproduction. Gene shuffling can create genetic diversity that facilitates adaptation to new environments, but theory shows that this effect is highly dependent on how different genes interact in determining the fitness of an organism. Using a large data set of fitness values based on HIV-1, we provide evidence that shuffling of genetic material indeed raises the level of genetic diversity, and as a result accelerates adaptation. Our results also propose genetic shuffling as a mechanism utilized by HIV to accelerate the evolution of multi-drug-resistant strains.

## Introduction

Recombination, here broadly defined as the shuffling of genetic material, is widespread in nature and occurs among a wide range of taxa, including most eukaryotes but also bacteria and viruses. It has long been believed that sex and recombination facilitate adaptation by increasing the genetic variance upon which natural selection can act [1]. However, recombination can also reduce variation if there is a preponderance of co-adapted allelic associations. This cost, referred to as recombination load, arises because recombination tends to unravel combinations of genes that are favored by selection, thus impeding adaptation [2]–[4]. Several hypotheses have been developed to account for the conditions under which recombination accelerates adaptation [4]–[8]. Epistasis-based hypotheses state that if beneficial mutations increase fitness less in combination than expected based on their individual effects (negative epistasis), recombination can accelerate adaptation by increasing genetic variance and thus enhancing the efficacy of selection [6], [7], [9]. In contrast, recombination is predicted to decelerate adaptation with positive as well as sign epistasis (i.e., when the direction of selection on an allele depends on the allelic status at other loci) [10]–[12]. According to another class of hypotheses, random genetic drift resulting from finite population size is believed to provide an advantage to recombination [4], [13]–[15]. This occurs because in finite populations, beneficial mutations are likely to occur on different backgrounds and compete with each other, thus reducing selection efficacy. Recombination alleviates this competition by bringing beneficial mutations together on the same background and consequently speeds up adaptation (the Fisher-Muller effect) [14]–[16].

Prior experimental studies have demonstrated that sex and recombination can facilitate adaptation [17]–[21]. Yet, our understanding of the costs and benefits of recombination during adaptation on empirical complex fitness landscapes is still limited. The structure of the underlying fitness landscape is a decisive factor for the effect of recombination on adaptation since it determines how natural selection creates non-random combinations of alleles. Although realistic fitness landscapes are believed to exhibit a complex structure characterized by intricate patterns of fitness interactions among genes, not much is known about the structure of large-scale fitness landscapes. This lack of knowledge hampered efforts to obtain insights into the effect of recombination on adaptation in realistic situations. To our knowledge, only one study explores the effect of recombination on an empirical fitness landscape [22], but this fitness landscape comprises only six loci, and thus, the results may not be generalizable to larger landscapes.

Recently, Hinkley et al. [23] have estimated a fitness landscape in HIV-1 for 1859 mutations, based on an in-vitro assay for viral replicative capacity. This empirical fitness landscape, by far the largest available empirical database characterizing epistatic interactions, allows us for the first time to scrutinize the impact of recombination on the evolutionary dynamics of an adapting population on a realistic fitness landscape. Through simulations on this fitness landscape, we examined the effect of recombination on adaptation under different conditions. We found benefits of recombination are sufficiently high to accelerate adaptation under a wide range of parameters. Our findings highlight the important evolutionary role of recombination in adaptation, and in particular, in HIV evolution.

## Results

**Figure 1:**
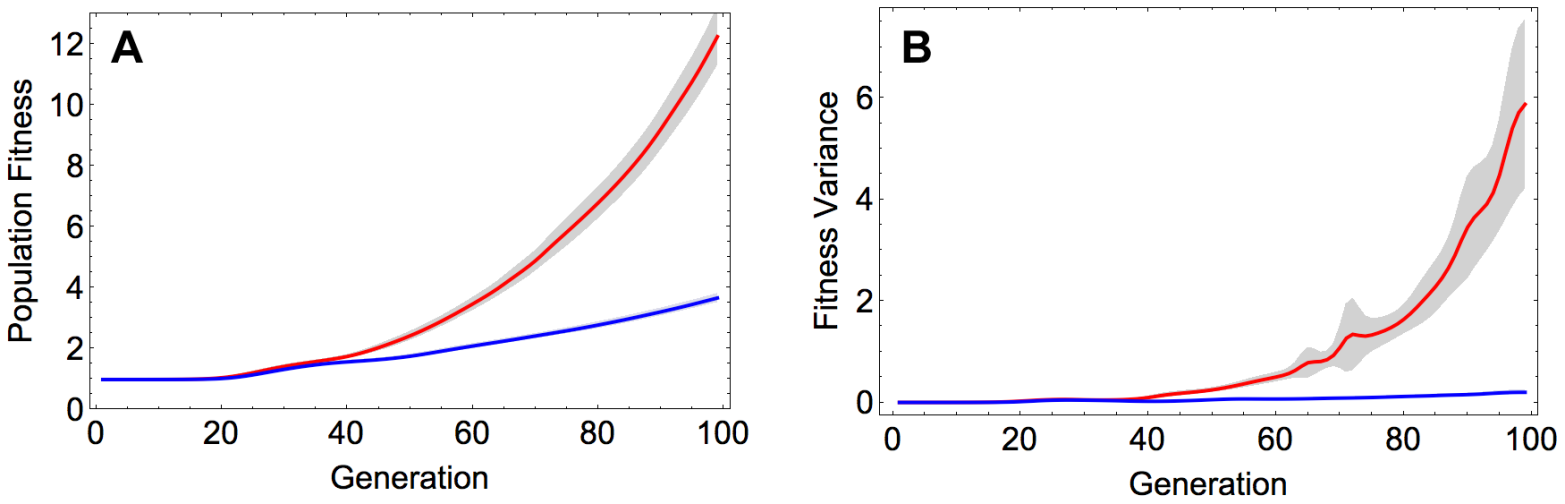
A) Population fitness and B) fitness variance in the recombining and non-recombining populations over time. Red and blue curves correspond to the mean value across 100 simulations in the recombining and non-recombining settings, respectively. Fitness and fitness variance values are normalized with the fitness of the reference sequence. The shaded regions show 95% confidence intervals. Parameters take values: 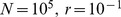 and 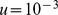.

Recombination was found to produce a substantial increase in the rate of adaptation (Figure 1A). This effect was robust with respect to the initial composition of the population, as it is observed not only when starting from the reference sequence but also when initializing the population with a random sequence (Figure S1 and Section S1 in Text S1). The acceleration of adaptation by recombination can be attributed to increased genetic variance in fitness, which in turn enhances the efficacy of natural selection, as proposed by the fundamental theorem of natural selection [14]. Indeed, we see a markedly stronger increase in genetic variance in fitness over time in the recombining, compared to the non-recombining population (Figure 1B). These results on population fitness are also in agreement with patterns of genetic diversity within the evolving populations: the recombining populations accumulate within-population diversity faster than the non-recombining populations (Figure S2A and Section S2 in Text S1), and they diverge faster from the initial sequence (Figure S2B and Section S2 in Text S1).

We also observed that with increasing recombination rate the fittest genotypes that emerged by the end of each simulation became more divergent from each other across replicate runs (Figure S3A and Section S3 in Text S1). However, it appears that this diversifying effect of recombination is primarily a consequence of the fact that recombination increases the rate at which the populations adapt and traverse the fitness landscape: when sequences are compared when the population reaches a certain threshold mean fitness, recombination has little effect on divergence (Figure S3B and Section S3 in Text S1). In other words, recombination accelerates adaptation but does not increase the number of evolutionary trajectories available to the populations in the course of adaptation.

We also considered how different drug treatments affect the impact of recombination on adaptation. To this end, we measured the effect of recombination using fitness landscapes obtained for 16 environments with different drug treatments. Our results indicate that the effect of recombination is markedly stronger in the presence of antiviral drugs compared to the drug-free environment (Figure S4 and Section S4 in Text S1), which appears to be due to the higher selection pressure in environments with drug treatments (results not shown).

**Figure 2:**
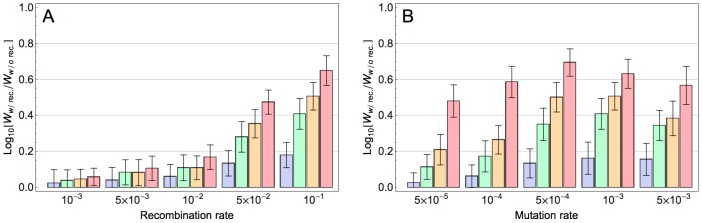
Effect of model parameters on the impact of recombination on adaptation. Each bar corresponds to the relative fitness of the recombining to the non-recombining population at generation 100, measured by taking the logarithm (base 10) of the ratio of mean fitness of the recombining population to that of the non-recombining population and averaged across 100 simulations. The blue, green, orange and red bars correspond to population sizes 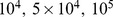 and 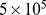, respectively. The error bars show the standard deviation of the difference between two log normal distributions (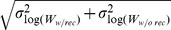). A) The effect of recombination rate at different population sizes for mutation rate: 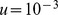. B) The effect of mutation rate at different population sizes for recombination rate: 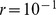. (The effect of recombination is still significant for populations larger than 10^3^ with this recombination rate; see Figure S5).

We next explored how the population size and mutation rate affect the extent to which recombination accelerates adaptation. Figure 2A indicates that, for a given population size, adaptation is already accelerated with moderate recombination rates and this effect increases monotonically with increasing recombination rates. In contrast, the effect of recombination depends non-monotonically on mutation rate (Figure 2B): whereas modest mutation rates enhance the accelerating effect of recombination on adaptation, at very high mutation rates this effect is reduced (see Discussion). Finally, our results indicate that with increasing population size, the accelerating effect of recombination becomes stronger (Figure 2A and S5 and Section S5 in Text S1). This is because with increasing population size, more beneficial mutations co-segregate in the population on different backgrounds and as a result the Fisher-Muller effect is enhanced. We expect that in very large populations this acceleration would become weaker again (because then all combinations of beneficial alleles would be present in the population even without recombination), but in the range of population sizes that was computationally feasible this was not observed. We also found the effect of recombination for populations with the same population mutation rate (population size×mutation rate) to be dependent on the population size: the effect is maximized for intermediate values of the population mutation rate and this maximum occurs at higher values for larger populations (Figure S6 and Section S6 in Text S1). This is presumably because for larger populations a higher mutation rate is required for multiple beneficial mutations to occur on the same background and thereby mitigate the Fisher-Muller effect.

**Figure 3:**
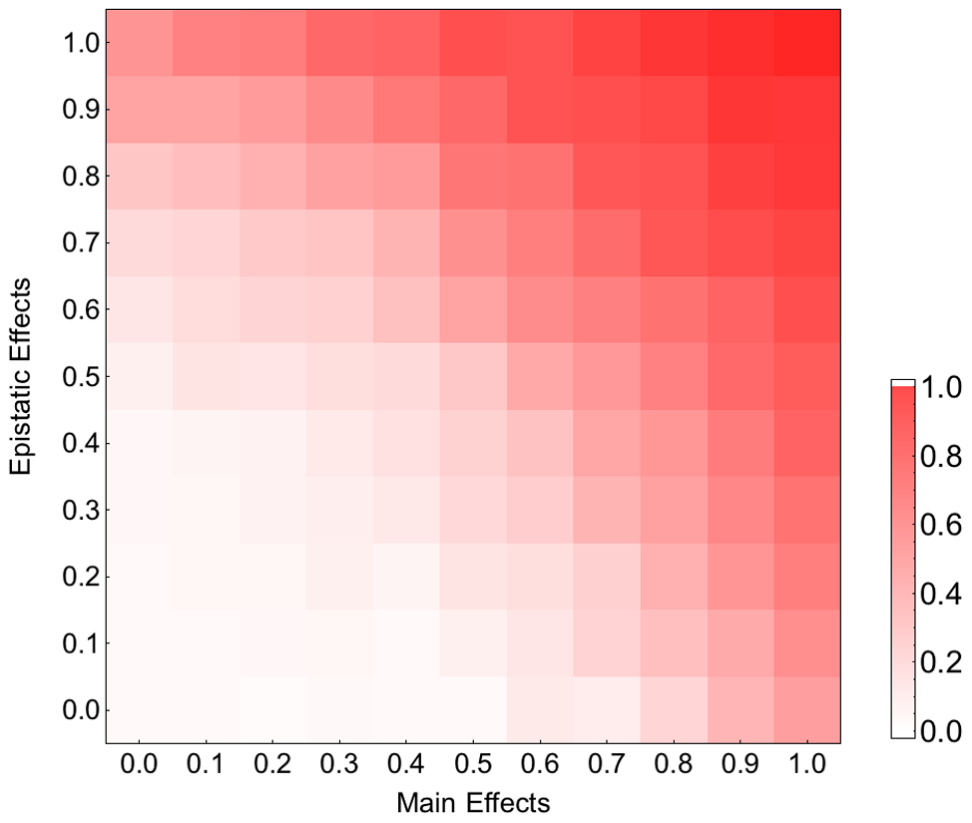
Contribution of the main and epistatic effects to the recombination effect on adaptation on the HIV-1 fitness landscape. The x and y axes show the values of the constants by which the elements of the main and epistatic effects, respectively, are multiplied in the hierarchical fitness landscape. The plot shows the logarithm (base 10) of the ratio of mean fitness of the recombining to that of the non-recombining populations at generation 100, averaged across 100 simulations. Parameters take values: 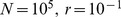 and 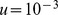.

Our fitness model incorporates both main and pairwise epistatic effects, both of which are known to influence the effect of recombination on the rate of adaptation. To assess the relative contribution of these effects, we simulated adaptation on fitness landscapes where, starting from the original **M** ^HL^ fitness matrix, we decreased the epistatic and main effects by varying amounts. Figure 3 indicates that both main effects and epistatic effects contribute to the acceleration of adaptation and that in combination these two effects appear to operate additively. Similar results were obtained for smaller recombination rates (Figure S7). We also found that both main effects and epistatic interactions increase the rate of adaptation and the effect of recombination becomes stronger with increasing adaptation rate (results not shown). These findings suggest that both main and epistatic effects can enhance selection. This seems to result in stronger interferences between arisen beneficial mutations and therefore a higher advantage of recombination (see Discussion).

**Figure 4:**
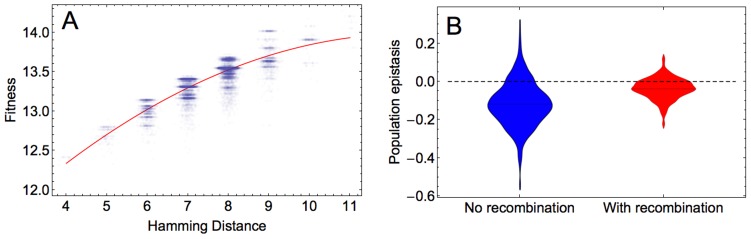
Population epistasis of the HIV-1 fitness landscape. A) An example of the log fitness plot against the Hamming distance between the reference sequence and sequences at generation 100 of one simulation for 20000 data points. Each point corresponds to one fitness value. To improve the visualization of the data on x axis, random values taken from a normal distribution with mean 0 and standard deviation 0.1 are added to the Hamming distances. The curvature of the red curved fitted to the data points defines population epistasis, which in this example is −0.0405. B) Distribution of population epistasis values across 500 simulations in the absence (blue) and presence (red) of recombination measured at generation 100. Parameters take values: 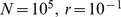 and 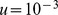.

We next determined the predominant form of epistasis in the adapting populations. To infer epistasis, one can use the fitness values of a set of sequences that are chosen irrespective of the composition of the evolving population and that therefore may not represent the sequences formed during adaptation (‘physiological epistasis’). Alternatively, only fitness values of sequences that are present in the adapting population can be utilized to estimate epistasis. This form of epistasis, referred to as population epistasis, provides a real time estimate of the epistasis that is responsible for generating the standing linkage disequilibrium in the population, and is therefore more accurate than physiological epistasis (see Discussion). We calculated population epistasis by regressing log fitness 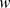 against Hamming distance, i.e., the number of sites where the corresponding sites are different between two sequences. The regression was done for 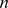 sequences that are present in the population at the end of simulation according to 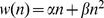, where Hamming distance 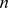 is measured relative to the reference sequence. The parameter 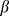, determining the curvature, is used as a measure of epistasis. Our results indicate that in the majority of simulations, population epistasis is significant (ANOVA test for comparison of a quadratic and a linear model, p≪0.001 for the simulations in Figure 4) and predominantly negative, indicating diminishing returns with each additional beneficial mutation in increasing fitness. Population epistasis in the recombining population becomes less negative on average than in the non-recombining population (Wilcoxon test for the significance of epistasis in Figure 4, p≪0.001 and for the significance of the difference between recombining and non-recombining simulations Figure 4B, p≪0.001). We obtained similar results for populations at other time points (results not shown).

Thus far, we considered the effect of recombination on adaptation by comparing evolving populations characterized by different recombination rates (including the absence of recombination). To examine whether recombination is selectively favored within an adapting population, we performed additional simulations in which we competed a resident non-recombining with an invading recombining genotype during adaptation. Figure S8A shows that the frequency of the recombining genotype gradually increases over time if the recombination rate is high enough 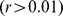. In accordance with our previous findings, the results of the invasion analysis demonstrate that the benefit of recombination is most pronounced for intermediate mutation rates (Figure S8B and S8C and Section S7 in Text S1).

## Discussion

Our results can be interpreted as support for the proposed accelerating role of recombination in the adaptive process through the Fisher-Muller effect. In our simulations, this effect seems to be sufficiently strong to outweigh potential costs of recombination. The Fisher-Muller effect is based on strong selective interference between beneficial mutations in an asexual (non-recombining) population. Previous mathematical models have provided important insights into how the strength of selection, mutation rate and population size affect selective interference and the Fisher-Muller effect, but these models have ignored epistatic interactions between mutations [24]–[30]. Our results demonstrate that both epistatic interactions and main fitness effects contribute to the accelerating effect of recombination. It is clear that this effect in the absence of epistatic interactions is due to the Fisher-Muller effect. Adding epistatic interactions to this model enhances the effect of recombination but the interpretation of why this occurs is challenging. On the one hand, this may be because the epistatic interactions increase the overall strength of selection and thereby produce stronger clonal interference, but on the other hand we cannot exclude explanations based purely on epistasis (see below).

One important determinant of the Fisher-Muller effect is the mutation rate. With a finite number of loci, an increasing mutation rate leads to a higher number of co-segregating beneficial mutations and this augments the Fisher-Muller effect. However, at very high mutation rates, it becomes increasingly likely that several beneficial mutations arise on the same genetic background so that recombination becomes less important (Figure 2B). A similar effect is expected for population size (with finite sites for beneficial mutations) but for the range of population sizes we examined here the effect of population size was monotonic.

In addition to the Fisher-Muller effect, recombination can also accelerate adaptation in the absence of random genetic drift when there is epistasis [9], [31]. Several studies have attempted to determine the prevailing form of epistasis in nature but have yielded inconsistent results: sometimes strong positive epistasis [32]–[36], negative epistasis [37], [38] or pervasive sign epistasis [39]–[42] was reported. The HIV-I fitness landscape is characterized by extensive epistatic interactions [23]. We demonstrated that population epistasis during adaptation is predominately negative. This result is in apparent contrast with the predominant positive epistasis in HIV-I sequences reported in Bonhoeffer *et al.* [33]. We think this discrepancy arises mainly because Bonhoeffer *et al.* estimated physiological epistasis, which is based only on the structure of fitness landscape itself and may be very different from population epistasis that we estimated here [43], [44]. The difference between the two estimates is that in determining population epistasis only the mutations that pass the sieve of natural selection are taken into account, whereas in measuring physiological epistasis all mutations are used indiscriminately. In addition to these different measures of epistasis, the two studies also differ in the way fitness was estimated. First, Bonhoeffer *et al.* [33] obtained fitness values from a much smaller data set than was used to estimate the fitness landscape in our study (9466 vs. 70,081 sequences). Second, in their study the main effects of a mutation and the epistatic effects for a given pair of mutations were calculated by averaging over the fitness effects of other mutations in the genetic background. By contrast, we obtained fitness using a predictive fitness model [23] that explicitly accounts for mutational effects in different genetic backgrounds during estimation of the fitness landscape, and therefore provides a more accurate estimate.

It is tempting to interpret the significant negative population epistasis that we observed as a support for the mechanism proposed by the mutational deterministic hypothesis, i.e. acceleration of adaptation through reduction of negative linkage disequilibria generated by negative epistasis. However, we would like to caution that it is very difficult to explain how population epistasis arises in our model and how it impacts the effect of recombination. For example, population epistasis on a complex fitness landscape can also be generated by variation in main fitness effects of mutations. The underlying causes of negative epistasis in our model and the extent to which it contributes to the accelerating effect of recombination (in isolation from the Fisher-Muller effect) is difficult to determine on a complex fitness landscape because deterministic models are not feasible. Therefore, we cannot exclude the possibility that stochastic benefits of recombination due to the Fisher-Muller effect may be sufficiently strong to override any direct effect of epistasis in our simulations, as reported by other studies [11], [45], [46].

Our study is based on an estimated fitness landscape and therefore the limitations of this approach should be taken into account while interpreting the results. First, in the fitness landscape that we used only main and pairwise epistatic effects were estimated but higher order fitness interactions (>2) were neglected. To accurately estimate higher orders epistasis, a much larger number of sequences with measured fitness values would be required. It is not clear what the strength of these higher-order interactions is and how they affect the impact of recombination. Second, although the accuracy of our fitness model (predicting 54.8% of the variance; see Methods) is acceptable as the only available fitness landscape, the predicted fitness landscape is yet to become more realistic by using a greater number of empirical fitness data. This is important as the estimated fitness values become increasingly unreliable for the regions in the sequence space far from the reference sequence due to the lack of data. To account for this problem, we focused on the population dynamics in the region of the fitness landscapes that is close to the reference sequence. Finally, the empirical fitness data used to predict the structure of the fitness landscape was obtained from an *in vitro* assay, and therefore might not completely correspond to *in vivo* fitness values.

It should also be noted that this study mainly examines the effect of recombination at the population level and does not address the evolution of recombination rate. One interesting extension of this work would be to incorporate variation in recombination rates in the model and study the spread of a recombination modifier gene in a non-recombining population. One problem with using the modifier approach in a realistic way with the current fitness model is that probably the best candidate for a recombination modifier gene in the HIV-1 genome is the reverse transcriptase gene [see 47], which is itself part of the fitness landscape and therefore changes during adaptation because of direct selection.

Our study relates to the debate over the advantage of recombination in retroviral, and in particular HIV, evolution. Recombination is believed by some to be beneficial because it generates genetic diversity to facilitate the development of multidrug resistance [48]–[50] or escape from host immune reaction [51]. Nonetheless, some studies have suggested that recombination has not evolved to facilitate adaptation but is a mere by-product of other mechanisms such as genomic organization [reviewed in 52], [ see also 45]. Unlike some prior studies [53]–[55], our model does not include any specific feature of HIV biology, such as viral dynamics during infection or specificities of recombination in HIV. Nonetheless, we believe that our findings are generic enough to highlight the potential role of recombination in accelerating HIV evolution.

This study utilizes data derived from a high-throughput fitness assay to address one of the long-standing questions in evolutionary biology. The advent of systems biology approaches made it possible to obtain a comprehensive picture of a large-scale fitness landscape. This serves as a framework for us to demonstrate that recombination has a substantial accelerating effect on adaptation on a realistic complex fitness landscape.

## Methods

### Fitness Model

Our model is based on a recently estimated fitness landscape of HIV-1 [23]. Briefly, to obtain this fitness landscape, the *in vitro* replicative capacity of 70,081 samples from HIV-1 subtype B infected individuals were measured and the corresponding amino acid sequences of the protease and partial sequences of the reverse transcriptase were obtained for all of these samples. This enabled estimation of the fitness effects of 1,857 single mutations and 257,536 pairs of mutations in these samples by fitting a fitness model to the data. This fitness model, as detailed in Hinkley et al. [23], invokes a generalized kernel ridge regression (GKRR) method to estimate the fitness effect of individual amino acid variants and the epistatic effects between variants.

Based on these results, we used the following fitness model to obtain fitness values for a given sequence:
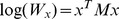



Here, the amino acid sequence **x** is a binary vector indicating the presence or absence of amino acid variants. **M** is a triangular matrix where an entry **M**_ii_ on the diagonal determines the main effects of the amino acid variant *i* and the off-diagonal entries **M**_ij_ (with 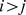) determine pairwise epistatic effects between variant *i* and *j*.

Higher order epistatic interactions were not considered. Note that the original model in Hinkley et al. [23] also includes an intercept term that gives the log fitness of a reference sequence (NL4-3) and that is added to Equation (1). However, since natural selection only depends on relative fitness in our model, this term was not considered in our simulation setting.

We used two different types of matrices **M** determining fitness. The first matrix type, **M** ^RL^, describing the ‘reference fitness landscape’, was obtained by Hinkley et al. [23] by estimating both main and epistatic effects simultaneously. This estimation was done in 16 different environments: one drug-free environment and 15 environments each characterized by the presence of a different antiretroviral drug. On average, this matrix predicts 54.8% of the variance in fitness across different environments. Unless stated otherwise, we use the fitness landscape in the drug-free environment as the reference fitness landscape in our simulations.

To obtain the second matrix type, **M** ^HL^, describing a ‘hierarchical fitness landscape’, two fitting steps were performed [56]. In the first step, **M** ^HL^ was estimated by assuming that there are only main effects (all off-diagonal elements set to zero), and in the second step, epistatic effects were estimated by fitting the residuals under the assumption that main effects are absent. This fitness landscape was obtained only for the drug free environment. Since the main and epistatic effects are estimated separately for these fitness landscapes, this approach allowed us to generate fitness landscapes where we could scale the magnitudes of main and epistatic effects and thus evaluate their relative contribution with respect to the effects of recombination. Hierarchical fitness landscapes with different magnitudes of epistatic effects were shown to provide accurate predictions of the reference fitness landscape [56].

For details about the estimation procedures, we refer to Hinkley et al. [23] and Kouyos et al. [56].

### Simulation Framework

We consider a discrete-time model based on the classic the Wright-Fisher model to simulate adaptive evolution under mutation, recombination and natural selection on the HIV-1 fitness landscape. The population consists of a constant number of 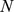 amino acid sequences, each of which contains the protease, as well as a partial reverse transcriptase, gene. Initially, this population is monomorphic, consisting only of 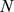 copies of a reference sequence (NL4-3).

In each generation, the new population is formed from the previous one through three steps. First, reproduction and selection are implemented through random sampling of 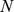 sequences, weighted according to the relative fitness value of each sequence. Second, to implement mutation events, 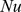 sequences are randomly chosen from the population with replacement (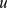 denotes the per genome mutation rate), thus allowing for several mutations per sequence. For 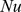
<1, this number is treated as a random number with mean 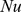. For each of these sequences, the allele at a randomly selected site for which there exists more than one possible variant is substituted with one of the other possible allelic variant. Amino acid variants at a given site that are not present in the data set used to estimate fitness are neglected. In the final step, selected sequences undergo homologous recombination. We denote the recombination rate by 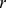. Here, 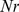 pairs of sequences are chosen randomly (without replacement) and for each of these pairs, a single crossover site at which the two parental sequences exchange genetic material is determined at random. Note that recombination may result in daughter sequences that are identical to the parental sequences if identical pieces are exchanged.

The simulations were run for 100 generations. This period is long enough for the population to adapt but the adapting population still remains in the proximity of the reference sequence, where due to the availability of empirical fitness data, the estimation of the fitness by our model is reliable. To examine the effect of recombination on adaptation, we computed the ratio of the logarithm (base 10) of the population mean fitness of a population evolving with recombination to that of a population evolving without recombination. In this case, finding a proper definition of error bars is not straightforward since the data in question are the logarithms of ratios. However, this logarithm can be written as a difference (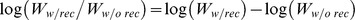), so that we can use the standard deviation of the difference between two log normal distributions, 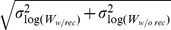, as error bars. This is justified because we found the log fitness values of sequences at generation 100 across 100 simulations to be normally distributed (for instance, non-significant Shapiro-Wilk and Anderson-Darling test to reject normal distribution for results in Figure 1, with p>0.05).

## Supporting Information

Figure S1Relative population fitness with vs. without recombination (as measured in Fig. 2) across 100 simulations over 100 generations, with 50 random initial populations and three mutation rates: A) 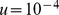 B) 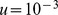 and C) 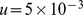. The curves show the mean value and the shaded region shows the 95% confidence interval. Other parameters take the values: 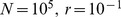.(TIFF)

Figure S2Effect of recombination on A) sequence variation and B) sequence divergence over time. A) Box plot of sequence variation over time in the recombining (red) and non-recombining (blue) populations as measured by the mean value of pairwise Hamming distances between sequences of a sample of 100 sequences in 100 simulations every 10 generations. The boxes give the interquartile range, the whiskers indicate the boundary of 1.5 times the interquartile range, and the points beyond that are outliers. B) Box plot of divergence of sequences from the initial population over time in the recombining (red) and non-recombining (blue) populations as measured by the mean values of the Hamming distances between a sample of 1000 sequences with the reference sequence for 100 simulations every 10 generations. The boxes, outliers and whiskers are defined the same as in panel A. Parameters values: 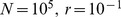 and 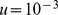.(TIFF)

Figure S3Hamming distance between the fittest sequences formed at A) generation 100 and B) generation at which the population mean fitness exceeds 3.16 times the fitness of the reference sequence across 100 simulations at different recombination rates. The outliers and whiskers are defined as in Figure S5. Parameters take values 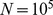 and 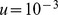.(TIFF)

Figure S4Effect of recombination on adaptation on fitness landscapes across different environments characterized by the absence of drugs or by the presence of a single antiretroviral drug. Color indicates drug-class (red: no drug; cyan: non-nucleoside reverse transcriptase inhibitor; blue: nucleoside analog reverse transcriptase inhibitor; green: protease inhibitor). Each bar shows the logarithm value (base 10) of the ratio of the average of population fitness values across 100 simulations in the recombining population to that in the non-recombining population at generation 100. The error bars are defined as in Figure 2. Parameter values: 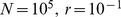 and 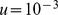.(TIFF)

Figure S5Effect of mutation rate on the impact of recombination on adaptation for smaller populations. Each bar shows the logarithm value (base 10) of the ratio of the average of population fitness values across 100 simulations in the recombining population to that in the non-recombining population at generation 100. The blue, green, yellow, orange and red bars correspond to population sizes 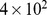, 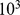, 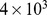, 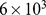 and 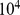, respectively. The error bars are defined as in Figure 2. Recombination rate: 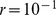.(TIFF)

Figure S6Effect of mutation rate on the impact of recombination on adaptation for population with the same population mutation rates (population size 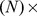
mutation rate 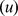). Each bar shows the logarithm value (base 10) of the ratio of the average of population fitness values across 100 simulations in the recombining population to that in the non-recombining population at generation 100. The blue, green and orange bars correspond to population sizes 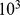, 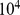 and 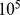, respectively. The error bars are defined as in Figure 2. Recombination rate: 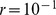.(TIFF)

Figure S7Contribution of the main and epistatic effects to the recombination effect on adaptation on the HIV-1 fitness landscape at two recombination rates A) 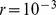 and B) 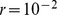. The x and y axes show the values of the constants by which the elements of the main and epistatic effects, respectively, are multiplied in the hierarchical fitness landscape. The plot shows the logarithm (base 10) of the ratio of the mean fitness of the recombining to that of the non-recombining populations at generation 100, averaged across 100 simulations. Parameters take the values: 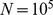 and 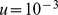.(TIFF)

Figure S8Invasion of the non-recombining population by the recombining type. The initial frequency of the recombining type is 1% of total the population size. The initial population is monomorphic, consisting only of the reference sequence. A) Frequency of the recombining type that invades a non-recombining population during adaptation, averaged across 500 simulations. The shaded region shows the 95% confidence interval. Parameters values: 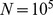, 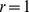 and 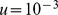 B), C), D) and E) Mean frequencies of the recombining population at generation 1000 across 500 simulations for different recombination rates A) 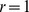 B) 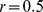 C) 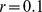 and D) 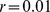. Each bar shows the mean final frequencies of the recombining population across 500 simulations at a different mutation rate. The error bars show the 95% confidence intervals. The dashed lines show the initial frequency of the recombining population (0.01). The population size was always set to 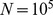.(TIFF)

Text S1 File contains information about the supplementary figures.
(PDF)

## References

[1] Weismann A (1904) The evolution theory. London: Edward Arnold.

[2] Otto SP, Gerstein AC (2006) Why have sex? The population genetics of sex and recombination. Biochem Soc Trans 34: 519–522. 16856849 10.1042/BST0340519

[3] Barton NH, Charlesworth B (1998) Why sex and recombination? Science 281: 1986–1990. 9748151

[4] Felsenstein J (1974) The evolutionary advantage of recombination. Genetics 78: 737–756. 4448362 10.1093/genetics/78.2.737 PMC1213231

[5] Maynard Smith JM (1988) Selection for recombination in a polygenic model–the mechanism. Genet Res 51: 59–63. 3366381 10.1017/s0016672300023958

[6] Charlesworth B (1993) Directional selection and the evolution of sex and recombination. Genet Res 61: 205–224. 8365658 10.1017/s0016672300031372

[7] Felsenstein J (1965) The effect of linkage on directional selection. Genetics 52: 349–363. 5861564 10.1093/genetics/52.2.349 PMC1210855

[8] Barton NH (1995) Linkage and the limits to natural selection. Genetics 140: 821–841. 7498757 10.1093/genetics/140.2.821 PMC1206655

[9] Kondrashov AS (1988) Deleterious mutations and the evolution of sexual reproduction. Nature 336: 435–440. 3057385 10.1038/336435a0

[10] de Visser JAGM, Elena SF (2007) The evolution of sex: empirical insights into the roles of epistasis and drift. Nature Reviews Genetics 8: 139–149. 10.1038/nrg1985 17230200

[11] Moradigaravand D, Engelstädter J (2012) The effect of bacterial recombination on adaptation on fitness landscapes with limited peak accessibility. PLoS Comput Biol 8: e1002735. 23133344 10.1371/journal.pcbi.1002735 PMC3487459

[12] Kondrashov FA, Kondrashov AS (2001) Multidimensional epistasis and the disadvantage of sex. Proc Natl Acad Sci U S A 98: 12089–12092. 11593020 10.1073/pnas.211214298 PMC59772

[13] Hill WG, Robertson A (1966) Effect of Linkage on Limits to Artificial Selection. Genetical Research 8: 269–294. 5980116

[14] Fisher RA (1930) The Genetical Theory Of Natural Selection USA: Oxford University Press.

[15] Muller HJ (1932) Some genetic aspects of sex. American Naturalist 66: 118–138.

[16] Kim Y, Orr HA (2005) Adaptation in sexuals vs. asexuals: Clonal interference and the Fisher-Muller model. Genetics 171: 1377–1386. 16020775 10.1534/genetics.105.045252 PMC1456838

[17] Rice WR, Chippindale AK (2001) Sexual recombination and the power of natural selection. Science 294: 555–559. 11641490 10.1126/science.1061380

[18] Colegrave N (2002) Sex releases the speed limit on evolution. Nature 420: 664–666. 12478292 10.1038/nature01191

[19] Poon A, Chao L (2004) Drift increases the advantage of sex in RNA bacteriophage Phi6. Genetics 166: 19–24. 15020402 10.1534/genetics.166.1.19 PMC1470714

[20] Goddard MR, Godfray HC, Burt A (2005) Sex increases the efficacy of natural selection in experimental yeast populations. Nature 434: 636–640. 15800622 10.1038/nature03405

[21] Becks L, Agrawal AF (2012) The Evolution of Sex Is Favoured During Adaptation to New Environments. Plos Biology 10: e1001317. 22563299 10.1371/journal.pbio.1001317 PMC3341334

[22] de Visser JAGM, Park SC, Krug J (2009) Exploring the Effect of Sex on Empirical Fitness Landscapes. American Naturalist 174: S15–S30. 10.1086/599081 19456267

[23] Hinkley T, Martins J, Chappey C, Haddad M, Stawiski E, et al (2011) A systems analysis of mutational effects in HIV-1 protease and reverse transcriptase. Nat Genet 43: 487–489. 21441930 10.1038/ng.795

[24] Desai MM, Fisher DS, Murray AW (2007) The speed of evolution and maintenance of variation in asexual populations. Current Biology 17: 385–394. 17331728 10.1016/j.cub.2007.01.072 PMC2987722

[25] Desai MM, Fisher DS (2007) Beneficial mutation selection balance and the effect of linkage on positive selection. Genetics 176: 1759–1798. 17483432 10.1534/genetics.106.067678 PMC1931526

[26] Weissman DB, Barton NH (2012) Limits to the rate of adaptive substitution in sexual populations. Plos Genetics 8: e1002740. 22685419 10.1371/journal.pgen.1002740 PMC3369949

[27] Sniegowski PD, Gerrish PJ (2010) Beneficial mutations and the dynamics of adaptation in asexual populations. Philosophical Transactions of the Royal Society B-Biological Sciences 365: 1255–1263. 10.1098/rstb.2009.0290 PMC2871819 20308101

[28] Neher RA (2013) Genetic Draft, Selective Interference, and Population Genetics of Rapid Adaptation. Annual Review of Ecology, Evolution, and Systematics, Vol 44 44: 195–215.

[29] Park SC, Krug J (2007) Clonal interference in large populations. Proceedings of the National Academy of Sciences of the United States of America 104: 18135–18140. 17984061 10.1073/pnas.0705778104 PMC2084309

[30] Park SC, Krug J (2013) Rate of Adaptation in Sexuals and Asexuals: A Solvable Model of the Fisher-Muller Effect. Genetics 195: 941–955. 23979572 10.1534/genetics.113.155135 PMC3813875

[31] Barton NH (1995) A General-Model for the Evolution of Recombination. Genetical Research 65: 123–144. 7605514 10.1017/s0016672300033140

[32] Trindade S, Sousa A, Xavier KB, Dionisio F, Ferreira MG, et al (2009) Positive epistasis drives the acquisition of multidrug resistance. Plos Genetics 5: e1000578. 19629166 10.1371/journal.pgen.1000578 PMC2706973

[33] Bonhoeffer S, Chappey C, Parkin NT, Whitcomb JM, Petropoulos CJ (2004) Evidence for positive epistasis in HIV-1. Science 306: 1547–1550. 15567861 10.1126/science.1101786

[34] He XL, Qian WF, Wang Z, Li Y, Zhang JZ (2010) Prevalent positive epistasis in Escherichia coli and Saccharomyces cerevisiae metabolic networks. Nature Genetics 42: 272–U120. 20101242 10.1038/ng.524 PMC2837480

[35] Maisnier-Patin S, Roth JR, Fredriksson A, Nystrom T, Berg OG, et al (2005) Genomic buffering mitigates the effects of deleterious mutations in bacteria. Nature Genetics 37: 1376–1379. 16273106 10.1038/ng1676

[36] Burch CL, Chao L (2004) Epistasis and its relationship to canalization in the RNA virus phi 6. Genetics 167: 559–567. 15238511 10.1534/genetics.103.021196 PMC1470902

[37] Khan AI, Dinh DM, Schneider D, Lenski RE, Cooper TF (2011) Negative epistasis between beneficial mutations in an evolving bacterial population. Science 332: 1193–1196. 21636772 10.1126/science.1203801

[38] Chou HH, Chiu HC, Delaney NF, Segre D, Marx CJ (2011) Diminishing Returns Epistasis Among Beneficial Mutations Decelerates Adaptation. Science 332: 1190–1192. 21636771 10.1126/science.1203799 PMC3244271

[39] Kvitek DJ, Sherlock G (2011) Reciprocal Sign Epistasis between Frequently Experimentally Evolved Adaptive Mutations Causes a Rugged Fitness Landscape. Plos Genetics 7: e1002056. 21552329 10.1371/journal.pgen.1002056 PMC3084205

[40] Silva RF, Mendonca SCM, Carvalho LM, Reis AM, Gordo I, et al (2011) Pervasive Sign Epistasis between Conjugative Plasmids and Drug-Resistance Chromosomal Mutations. Plos Genetics 7: e1002181. 21829372 10.1371/journal.pgen.1002181 PMC3145620

[41] Weinreich DM, Delaney NF, DePristo MA, Hartl DL (2006) Darwinian evolution can follow only very few mutational paths to fitter proteins. Science 312: 111–114. 16601193 10.1126/science.1123539

[42] Franke J, Klozer A, de Visser JA, Krug J (2011) Evolutionary accessibility of mutational pathways. PLoS Comput Biol 7: e1002134. 21876664 10.1371/journal.pcbi.1002134 PMC3158036

[43] Kouyos RD, Silander OK, Bonhoeffer S (2007) Epistasis between deleterious mutations and the evolution of recombination. Trends Ecol Evol 22: 308–315. 17337087 10.1016/j.tree.2007.02.014

[44] Kouyos RD, Otto SP, Bonhoeffer S (2006) Effect of varying epistasis on the evolution of recombination. Genetics 173: 589–597. 16547114 10.1534/genetics.105.053108 PMC1526506

[45] Otto SP, Barton NH (2001) Selection for recombination in small populations. Evolution 55: 1921–1931. 11761054 10.1111/j.0014-3820.2001.tb01310.x

[46] Keightley PD, Otto SP (2006) Interference among deleterious mutations favours sex and recombination in finite populations. Nature 443: 89–92. 16957730 10.1038/nature05049

[47] Onafuwa-Nuga A, Telesnitsky A (2009) The remarkable frequency of human immunodeficiency virus type 1 genetic recombination. Microbiol Mol Biol Rev 73: 451–480 Table of Contents 19721086 10.1128/MMBR.00012-09 PMC2738136

[48] Gu Z, Gao Q, Faust EA, Wainberg MA (1995) Possible involvement of cell fusion and viral recombination in generation of human immunodeficiency virus variants that display dual resistance to AZT and 3TC. J Gen Virol 76 Pt 10: 2601–2605. 7595365 10.1099/0022-1317-76-10-2601

[49] Kellam P, Larder BA (1995) Retroviral Recombination Can Lead to Linkage of Reverse-Transcriptase Mutations That Confer Increased Zidovudine Resistance. Journal of Virology 69: 669–674. 7529334 10.1128/jvi.69.2.669-674.1995 PMC188627

[50] Moutouh L, Corbeil J, Richman DD (1996) Recombination leads to the rapid emergence of HIV-1 dually resistant mutants under selective drug pressure. Proc Natl Acad Sci U S A 93: 6106–6111. 8650227 10.1073/pnas.93.12.6106 PMC39197

[51] Malim MH, Emerman M (2001) HIV-1 sequence variation: Drift, shift, and attenuation. Cell 104: 469–472. 11239404 10.1016/s0092-8674(01)00234-3

[52] Simon-Loriere E, Holmes EC (2011) Why do RNA viruses recombine? Nature Reviews Microbiology 9: 617–626. 21725337 10.1038/nrmicro2614 PMC3324781

[53] Fraser C (2005) HIV recombination: what is the impact on antiretroviral therapy? Journal of the Royal Society Interface 2: 489–503. 10.1098/rsif.2005.0064 PMC1618498 16849208

[54] Bretscher MT, Althaus CL, Muller V, Bonhoeffer S (2004) Recombination in HIV and the evolution of drug resistance: for better or for worse? Bioessays 26: 180–188. 14745836 10.1002/bies.10386

[55] Kouyos RD, Fouchet D, Bonhoeffer S (2009) Recombination and drug resistance in HIV: Population dynamics and stochasticity. Epidemics 1: 58–69. 21352751 10.1016/j.epidem.2008.11.001

[56] Kouyos RD, Leventhal GE, Hinkley T, Haddad M, Whitcomb JM, et al (2012) Exploring the complexity of the HIV-1 fitness landscape. Plos Genetics 8: e1002551. 22412384 10.1371/journal.pgen.1002551 PMC3297571

