# Supplementary figures and images for: Recombination Accelerates Adaptation on a Large-Scale Empirical Fitness Landscape in HIV-1
Source: PLoS Genet. 2014 Jun 26;10(6):e1004439. doi: 10.1371/journal.pgen.1004439 (PMC4072600; doi:10.1371/journal.pgen.1004439)

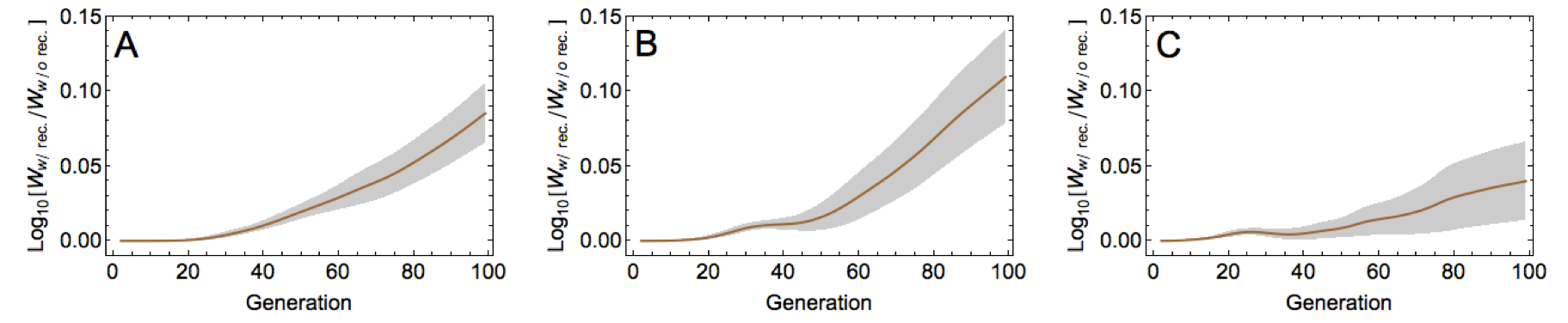

Supplement: Figure S1 — Relative population fitness with vs. without recombination (as measured in Fig. 2) across 100 simulations over 100 generations, with 50 random initial populations and three mutation rates: A) B) and C) . The curves show the mean value and the shaded region shows the 95% confidence interval. Other parameters take the values: . (TIFF) [file pgen.1004439.s001.tiff]

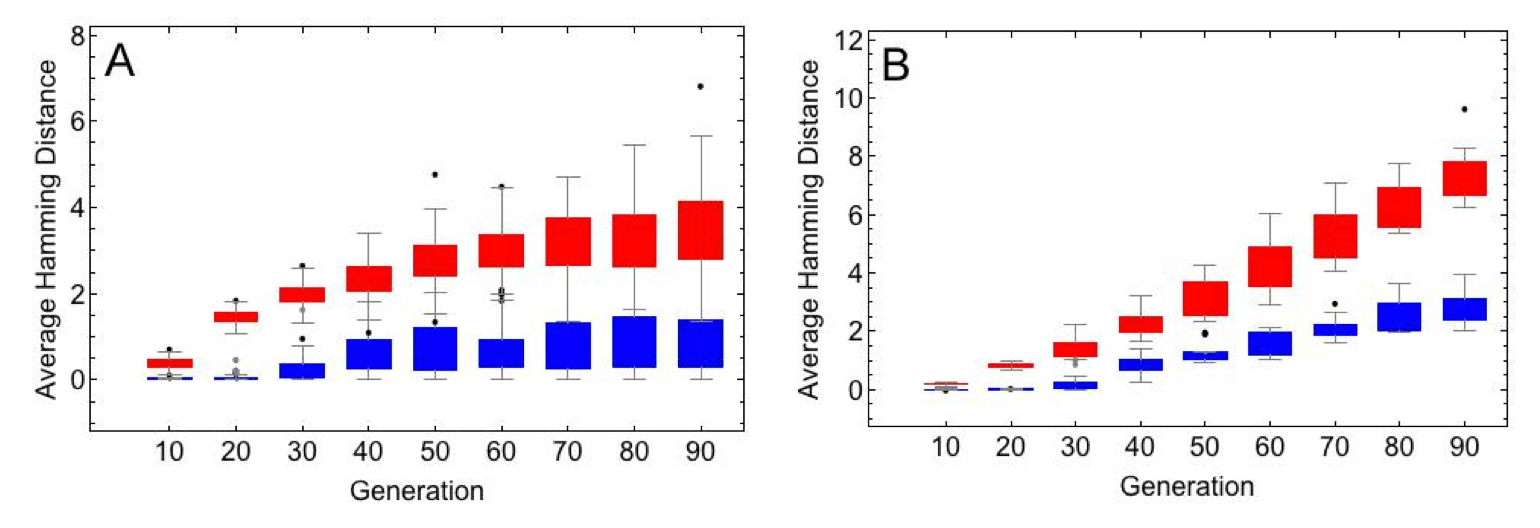

Supplement: Figure S2 — Effect of recombination on A) sequence variation and B) sequence divergence over time. A) Box plot of sequence variation over time in the recombining (red) and non-recombining (blue) populations as measured by the mean value of pairwise Hamming distances between sequences of a sample of 100 sequences in 100 simulations every 10 generations. The boxes give the interquartile range, the whiskers indicate the boundary of 1.5 times the interquartile range, and the points beyond that are outliers. B) Box plot of divergence of sequences from the initial population over time in the recombining (red) and non-recombining (blue) populations as measured by the mean values of the Hamming distances between a sample of 1000 sequences with the reference sequence for 100 simulations every 10 generations. The boxes, outliers and whiskers are defined the same as in panel A. Parameters values: and . (TIFF) [file pgen.1004439.s002.tiff]

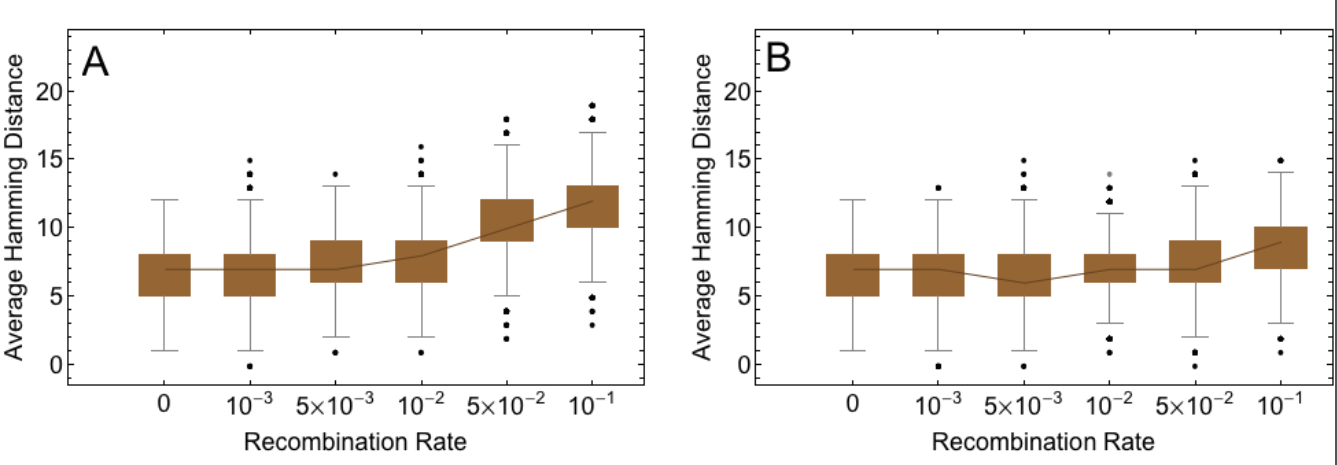

Supplement: Figure S3 — Hamming distance between the fittest sequences formed at A) generation 100 and B) generation at which the population mean fitness exceeds 3.16 times the fitness of the reference sequence across 100 simulations at different recombination rates. The outliers and whiskers are defined as in Figure S5. Parameters take values and . (TIFF) [file pgen.1004439.s003.tiff]

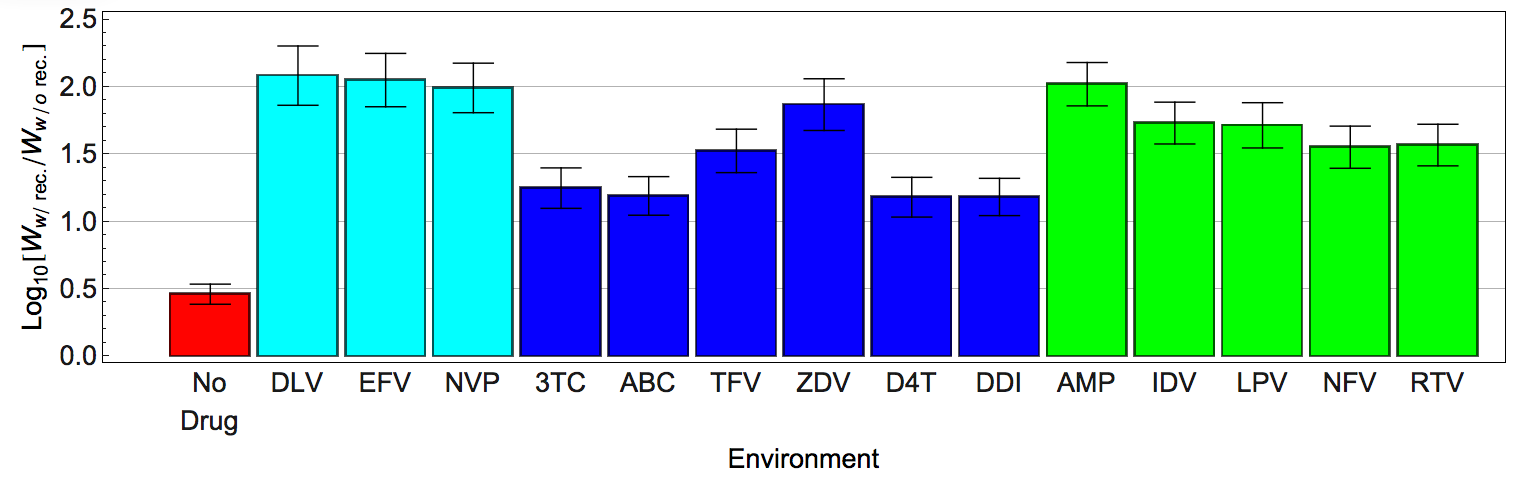

Supplement: Figure S4 — Effect of recombination on adaptation on fitness landscapes across different environments characterized by the absence of drugs or by the presence of a single antiretroviral drug. Color indicates drug-class (red: no drug; cyan: non-nucleoside reverse transcriptase inhibitor; blue: nucleoside analog reverse transcriptase inhibitor; green: protease inhibitor). Each bar shows the logarithm value (base 10) of the ratio of the average of population fitness values across 100 simulations in the recombining population to that in the non-recombining population at generation 100. The error bars are defined as in Figure 2. Parameter values: and . (TIFF) [file pgen.1004439.s004.tiff]

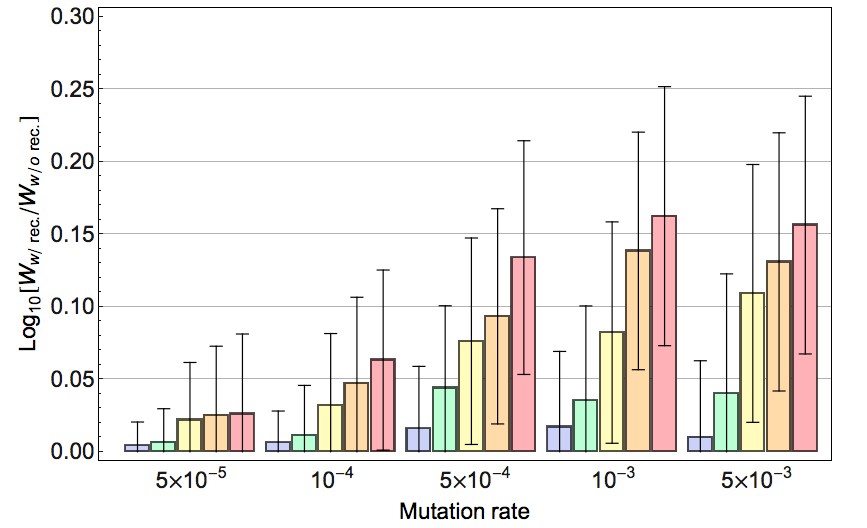

Supplement: Figure S5 — Effect of mutation rate on the impact of recombination on adaptation for smaller populations. Each bar shows the logarithm value (base 10) of the ratio of the average of population fitness values across 100 simulations in the recombining population to that in the non-recombining population at generation 100. The blue, green, yellow, orange and red bars correspond to population sizes , , , and , respectively. The error bars are defined as in Figure 2. Recombination rate: . (TIFF) [file pgen.1004439.s005.tiff]

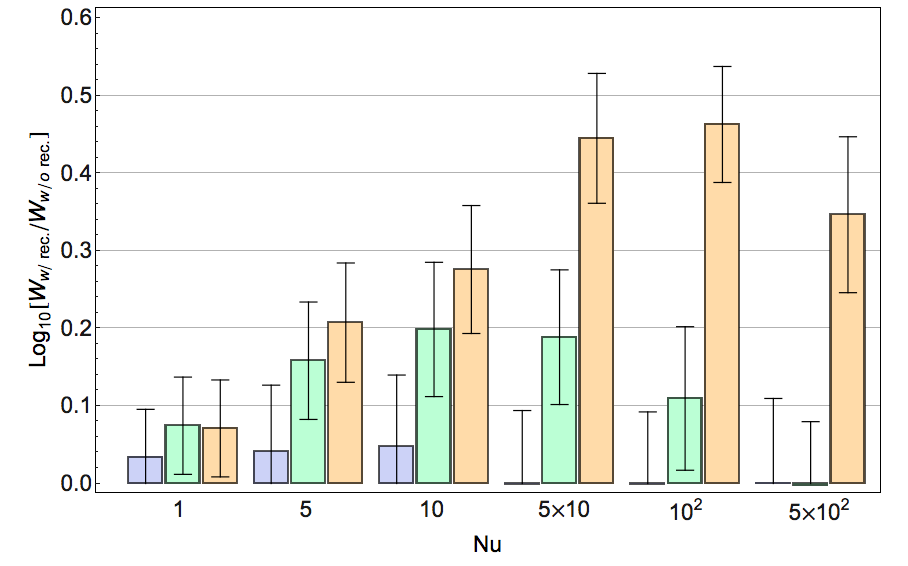

Supplement: Figure S6 — Effect of mutation rate on the impact of recombination on adaptation for population with the same population mutation rates (population size mutation rate ). Each bar shows the logarithm value (base 10) of the ratio of the average of population fitness values across 100 simulations in the recombining population to that in the non-recombining population at generation 100. The blue, green and orange bars correspond to population sizes , and , respectively. The error bars are defined as in Figure 2. Recombination rate: . (TIFF) [file pgen.1004439.s006.tiff]

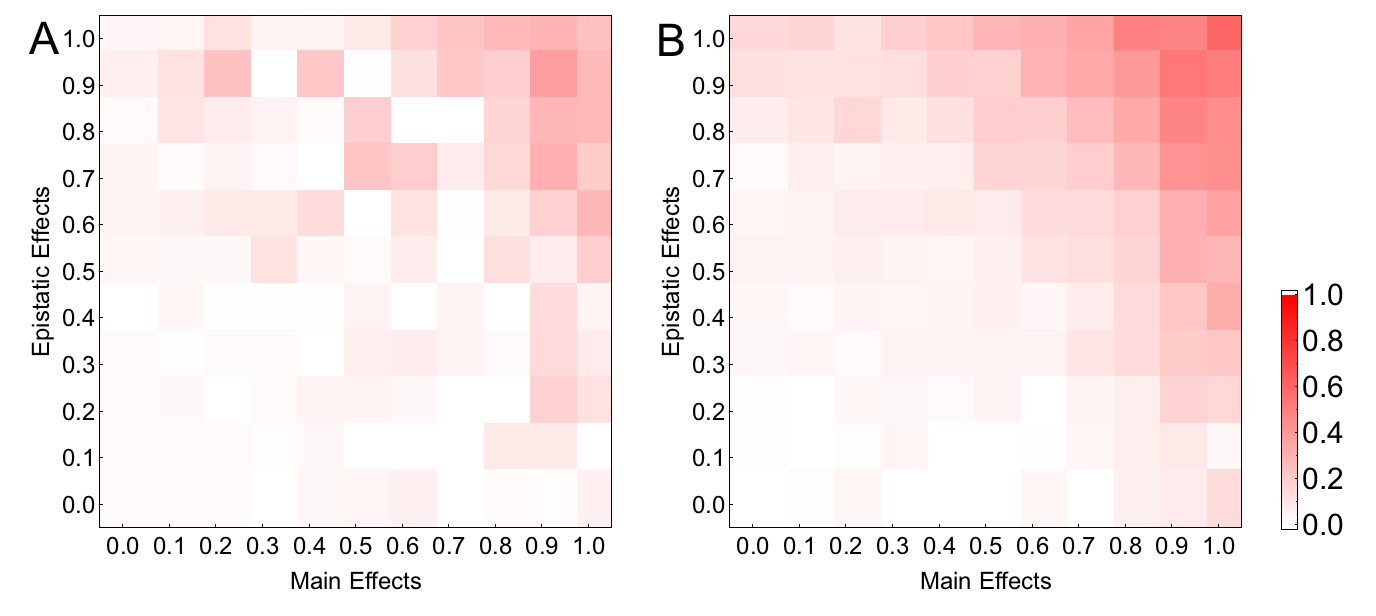

Supplement: Figure S7 — Contribution of the main and epistatic effects to the recombination effect on adaptation on the HIV-1 fitness landscape at two recombination rates A) and B) . The x and y axes show the values of the constants by which the elements of the main and epistatic effects, respectively, are multiplied in the hierarchical fitness landscape. The plot shows the logarithm (base 10) of the ratio of the mean fitness of the recombining to that of the non-recombining populations at generation 100, averaged across 100 simulations. Parameters take the values: and . (TIFF) [file pgen.1004439.s007.tiff]

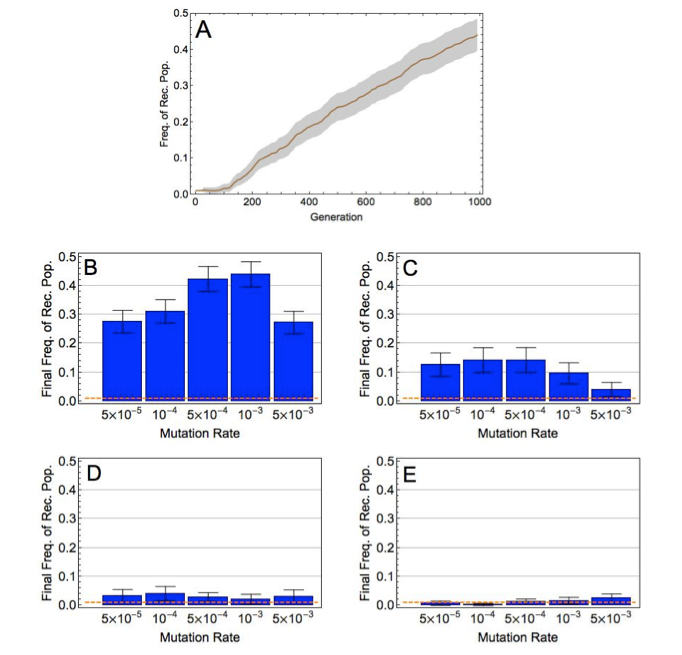

Supplement: Figure S8 — Invasion of the non-recombining population by the recombining type. The initial frequency of the recombining type is 1% of total the population size. The initial population is monomorphic, consisting only of the reference sequence. A) Frequency of the recombining type that invades a non-recombining population during adaptation, averaged across 500 simulations. The shaded region shows the 95% confidence interval. Parameters values: , and B), C), D) and E) Mean frequencies of the recombining population at generation 1000 across 500 simulations for different recombination rates A) B) C) and D) . Each bar shows the mean final frequencies of the recombining population across 500 simulations at a different mutation rate. The error bars show the 95% confidence intervals. The dashed lines show the initial frequency of the recombining population (0.01). The population size was always set to . (TIFF) [file pgen.1004439.s008.tiff]
