# Supplementary material for: Recombination Accelerates Adaptation on a Large-Scale Empirical Fitness Landscape in HIV-1
Source: PLoS Genet. 2014 Jun 26;10(6):e1004439. doi: 10.1371/journal.pgen.1004439 (PMC4072600; doi:10.1371/journal.pgen.1004439)
Supplement: Text S1 — File contains information about the supplementary figures. (PDF) [file pgen.1004439.s009.pdf]

Text S1

Recombination accelerates adaptation on a large-scale empirical fitness landscape in HIV-1

Danesh Moradigaravand, Roger Kouyos, Trevor Hinkley, Mojgan Haddad, Christos J Petropoulos, Jan Engelstädter & Sebastian Bonhoeffer

S1. The impact of initial population composition on the effect of recombination on adaptation

To examine whether the accelerating effect of recombination holds also for initial populations consisting of sequences other than NL4-3, we generated 50 random initial populations each consisting of identical copies of one sequence. These sequences were generated by randomly drawing one of the available allelic variants for polymorphic sites. We then performed simulations in the presence and absence of recombination. For each time point, the ratio of the average of population fitness values of the recombining to that of the non-recombining setting across 100 simulations was calculated and this was repeated for all initial populations. Figure S1 indicates that recombination accelerates adaptation independently of the composition of the initial population. Similar to Figure 2, the effect of recombination is also non-monotonic with increasing mutation rate for other initial populations (Figure S1B, S1C).

S2. The effect of recombination on population genetic diversity and divergence.

We compared within-population genetic diversity of the recombining population with that of the non-recombining population. To this end, we took a sample from the recombining and non-recombining populations every 10 generations during adaptation. We then calculated the mean of the pairwise Hamming distances, i.e., the number of sites where the corresponding alleles are different between two sequences, between these randomly sampled sequences. Figure S2A shows that sequence diversity increases over time in both populations but is higher in the recombining population. This is in accordance with the higher variance in fitness in the recombining population, reported in Figure 1B.

To evaluate the effect of recombination on the rate at which populations diverge from the initial population over time, we measured the Hamming distance between all sequences in the samples from the reference sequence. Figure S2B reveals that recombination causes the population to diverge faster over time.

S3. The effect of recombination on the ability of populations to explore the fitness landscape.

We first calculated the Hamming distance between the fittest sequences formed after 100 generations across 100 simulations in populations with increasing recombination rate. We next assessed whether recombination increases the ability of populations to take different trajectories during adaptation across replicate runs. We addressed this by examining the diversity between sequences formed when the recombining and non-recombining populations had adapted to the same extent. More precisely, we measured the Hamming distance between the fittest genotypes formed across 100 simulations at the time when population mean fitness first exceeds a certain threshold (3.16 times the fitness of the reference sequence). This helps us to disentangle the accelerating and the diversifying potential of recombination.

Our results (Figure S3B) do not show a significant effect of recombination on the divergence of the fittest sequences formed after the recombining and non-recombining populations proceed up to the same fitness threshold. (This also holds when we measured sequence divergence between the fittest sequences formed after a certain threshold in the recombining to those in the non-recombining populations; results not shown.) This indicates that recombination does not increase the number of routes that the population takes during adaptation on the fitness landscape but only accelerates adaptation.

S4. The effect of recombination on adaptation on fitness landscapes in different drug environments.

As mentioned in the Methods, the fitness assay was conducted in one drug-free environment and 15 environments with different antiretroviral drugs. Figure S4 shows the effect of recombination on adaptation on fitness landscapes in environments without drug treatment and with drugs of 3 main classes of antiretroviral drugs: protease inhibitors (PI), nucleoside reverse transcriptase inhibitors (NRTI), and non-nucleoside reverse transcriptase inhibitors (NNRTI). Recombination effects are much stronger in the presence of drugs, suggesting that the differences in the characteristics of landscapes, as discussed in Kouyos et al. [1], influence the impact of recombination. We also observed that in the fitness landscapes corresponding to the NNRTI and PI drug classes, where recombination has the strongest effect, the selection pressure on allelic variants is, on average, slightly higher (not shown). The higher selection pressure may intensify the interference between beneficial alleles during adaptation and consequently lead to the stronger effect of recombination on adaptation in these environments compared to the free-drug environment. The selection pressure, however, does not seem to be strong enough to remove polymorphism in the population and thus make recombination unimportant.

The result that higher levels of stress – in this case drug treatments – lead to an increased advantage of recombination is also consistent with the idea of selection for stress-induced recombination. Indeed, a substantial advantage of fitness-associated recombination was previously demonstrated under adaptation on both smooth and rugged landscapes [2,3]. However, whether or not strains with stress-induced recombination would be selected for on our HIV-1 fitness landscapes needs to be explored in future studies that explicitly compete such strains with strains characterized a constant recombination rate.

S5. Further exploration of the parameter space for smaller population sizes.
In this section, we present the results of the simulations for populations smaller than 10^4. As shown by Figure S5, the effect of recombination becomes insignificant for population sizes equal to or smaller than 10^3 or mutation rates smaller than 10^{-4} where there are not sufficient number of interfering beneficial mutations in the population.

S6. The effect of recombination for populations with the same population mutation rates.

In this section, we present the results of simulations of populations with the same values for the population mutation rate, i.e. the product of population size and mutation rate. Figure S6 shows that the impact of recombination on the rate of adaptation is maximized at a certain intermediate value of Nu. This is because with small Nu only few beneficial mutations co-segregate in the population whereas with very large Nu the probability that multiple beneficial mutations arise on the same background becomes higher. Thus, the Fisher-Muller effect is undermined for both small and large values of Nu. However, the value of Nu where recombination has the strongest effect of accelerating adaptation increases with N, indicating that not only the number of new mutations that enter the population every generation is important but also the amount of random genetic drift.

S7. Invasion of non-recombining population by recombining type

In this section, we examine the invasion of a recombining type into a resident non-recombining population in the course of adaptation. We assume that recombination occurs only between individuals of the recombining type (with probability r) but not between the non-recombining and the recombining type. Figure S8A shows that on average, the frequency of the recombining type increases over time for large recombination rates. In agreement with Figure 2, we found that the recombining type becomes most invasive at intermediate mutation rates and high recombination rates (Fig. S8B-E). However, the effect of recombination is still pronounced for recombination rates as low as 0.001.

References

1. Kouyos RD, Leventhal GE, Hinkley T, Haddad M, Whitcomb JM, et al. (2012) Exploring the complexity of the HIV-1 fitness landscape. Plos Genetics 8: e1002551.
2. Hadany L, Beker T (2003) On the evolutionary advantage of fitness-associated recombination. Genetics 165: 2167-2179.
3. Hadany L, Beker T (2003) Fitness-associated recombination on rugged adaptive landscapes. J Evol Biol 16: 862-870.
